# Psychological Support for Young Adults with Down Syndrome: Dohsa-Hou Program for Maladaptive Behaviors and Internalizing Problems

**DOI:** 10.3389/fpsyg.2017.01504

**Published:** 2017-09-01

**Authors:** Haruo Fujino

**Affiliations:** ^1^Department of Special Needs Education, Oita University Oita, Japan; ^2^Graduate School of Human Sciences, Osaka University Osaka, Japan

**Keywords:** Down syndrome, Dohsa-hou, mind-body therapies, social withdrawal, regression, depression, mental disorders

## Abstract

Psychological and psychiatric dysfunction is a major problem in a substantial proportion of young adults with Down syndrome. Some patients develop psychiatric issues, such as depressive, obsessive-compulsive, or psychotic-like disorders, in their late adolescence or young adulthood. Furthermore, these individuals may experience moderate to severe emotional and psychological distress. Development of a psychosocial treatment to address these issues is needed in addition to psychotropic medication. The current study reports two cases of young adults with Down syndrome, who presented psychiatric symptoms and marked disruption in their daily lives. These individuals participated in a Dohsa-hou treatment program. Following treatment, adaptive levels, maladaptive behaviors, and internalizing problems were evaluated by the Vineland Adaptive Behavior Scales-II. Participants showed improvement in maladaptive behaviors and internalizing problems; however, improvement in these areas may be influenced by baseline severity of the problems. This case report suggests that Dohsa-hou could be an effective therapeutic approach for maladaptive and internalizing problems in adults with Down syndrome.

## Introduction

Down syndrome is one of the most common condition caused by autosomal chromosomal abnormality, which occurs in approximately 1 in 800 newborn children (Shin et al., 2009). A majority of individuals with Down syndrome have mild to severe intellectual disabilities, as well as disabilities in social functioning (Bertoli et al., 2011; Covelli et al., 2015). Additionally, psychiatric problems can affect the adaptation of these individuals.

The estimated prevalence of psychiatric disorders (e.g., depressive, obsessive-compulsive, and psychotic-like disorders) is 18 to 38% in individuals with Down syndrome; although this prevalence rate could be affected by diagnostic criteria and methods used to evaluate psychiatric symptoms (Capone et al., 2006; Maatta et al., 2006). Difficulty in diagnosing comorbid disorders are often related to the disabled intellectual functioning typically observed in these individuals (Maatta et al., 2006; Channell et al., 2015). Common symptoms in depressive disorder include depressed mood, decreased interest, psychomotor slowing, sleep disturbance, and weight change. Conversely, social withdrawal and compulsive behavior (routinized behavior) are associated features in depressed adults with Down syndrome (Sutor et al., 2006).

A significant proportion of young adults with Down syndrome have also been described to experience suffering from functional deterioration in early adulthood. The psychiatric presentation of this deterioration is similar to depressive disorders or obsessive-compulsive disorders. However, the underlying cause of the condition may be associated with particular medical conditions (e.g., the beginnings of neurofibrillary tangles), or preceding psychological stressors (Prasher, 2002; Capone et al., 2006; Worley et al., 2015). Such circumstances may be a result of an unclear understanding of the mechanism of deterioration. Regardless of the specific cause, daily self-care routines can deteriorate, requiring assistance or frequent prompting by the caregiver. As such, these types of conditions markedly impact the lives of the individuals, as well as their family.

Despite the importance of emotional and behavioral problems in Down syndrome, intervention and treatment studies for these problems are limited. Psychosocial treatment may be useful for these issues, but psychotropic medication is the most frequently used approach for psychiatric symptoms and behavioral problems. Several studies have reported beneficial effects on their problems to some extent (Geldmacher et al., 1997; Jacobs et al., 2016; Tamasaki et al., 2016). However, psychosocial treatments for such problems in adults with Down syndrome are rarely reported (Vicari et al., 2013).

Dohsa-hou is a psychosocial treatment approach, which has been used in clinical practice mainly in Japan and East Asian countries (Naruse, 1973; Dadkhah, 1998; Imura and Chervenkova, 2016), which was developed by Naruse and his colleagues from 1970s (Naruse, 1992). Early practice of Dohsa-hou had been developed as physical and psychological support for children with cerebral palsy. Later, Dohsa-hou was used as psychological rehabilitation in various disorders including Down syndrome (Naruse, 1995; Imura and Chervenkova, 2016). This approach uses body movement, bodily feeling, and the experience of relaxation and body movement as a means of therapeutic intervention and communication (Naruse, 1995; Fujino, 2012). Standing tasks, to keep balance and to stand upright, are also used in Dohsa-hou because gross motor function is also a major problem in various disorders that affect physical functions. Although clinicians have described case reports of Dohsa-hou in children and adolescents with Down syndrome, suggesting improvement in psychological and social functioning (Tanaka, 1988, 1992; Satoh, 1992; Sasagawa et al., 2000), more research is needed to clarify clinical utility of this technique in young adults with Down syndrome. The current case report provides preliminary evidence for the effectiveness of a psychosocial approach, based on Dohsa-hou, on maladaptive behaviors and internalizing problems.

## Background

I describe here the background and chief complaints of two adult men with Down syndrome. These individuals participated in a short-term Dohsa-hou treatment program to improve their emotional and behavioral problems. Personal and social skills needed for everyday living were evaluated using the Vineland Adaptive Behavior Scales-II (Vineland-II) (Sparrow et al., 2005). The Vineland-II provides an adaptive behavior composite score as a measure of the overall adaptive level of functioning, which is derived from standard scores of Communication, Daily living skills, and Socializing domains. A Maladaptive Behavior Index provides measurement of maladaptive (symptomatic) behaviors exhibited by individuals. Internalizing score was also used. This study was carried out in accordance with the recommendations of the World Medical Association’s Declaration of Helsinki, with written informed consent from the guardians of all participants. The protocol was approved by the Research Ethics Review Board of Graduate School of Human Sciences at Osaka University. Names and identifying details have been changed to protect privacy of the participants.

### Case Background and Presenting Complaints

Ken, a 24-year-old man with Down syndrome, came to receive treatment with his mother. He lived in a group home, where he received living assistance, and returned on weekends to the house where his mother lived. He did not actively engage with people, nor did he appear to reject others who approached him. His awareness of the many things that he cannot do, compared to his peers, had negatively affected his self-esteem. For this reason, his relationships with those in his environment had recently begun to weaken. Furthermore, he also began to find enjoyment in fewer activities in his daily life. He was rarely proactive about performing his duties in his workplace or in other settings; instead, he passively did what he was told to do. In addition, his mother was concerned that when the two of them were together, he left decisions to her and tended to rely on her for assistance, even with tasks he was capable of completing by himself. He was able to speak in simple two-word sentences and communicated his intentions on his own. At that time, he was not receiving treatment from any medical institution.

His Vineland-II evaluation showed a higher score on the Socialization subscale compared to his scores on the Communication and Daily Living Skills subscales; overall, his general adaptation level was low (**Table 1**).

**Table 1:** Assessment of adaptive level of participants evaluated by the Vineland Adaptive Behavior Scales-II.

Domain/	Ken	Adaptive	Atsushi	Adaptive
subdomain	score	level	score	level
Adaptive behavior composite	20	Low	20	Low
Communication	20	Low	20	Low
Daily living skills	20	Low	20	Low
Socialization	32	Low	20	Low
Subdomain scores				
Receptive	7	Low	2	Low
Expressive	1	Low	1	Low
Written	1	Low	1	Low
Personal	2	Low	1	Low
Domestic	5	Low	1	Low
Community	1	Low	1	Low
Interpersonal relationships	7	Low	2	Low
Play and leisure time	1	Low	1	Low
Coping skills	2	Low	1	Low

Atsushi, a 30-year-old man with Down syndrome, came to receive treatment with his mother. He lives at home with his family. He rarely approached others spontaneously. In social situations, such as group activities, he was often disagreeable and reluctant to participate. He communicated his intentions to others using two to three words, such as “uh-huh” and “yes” (for agreement) and “ugh” (for objection). He often engaged in self-stimulatory behaviors, such as vocalizing and swaying back and forth, and showed little reaction to, or engagement with his surroundings. He also developed compulsive behaviors during meals and when moving between rooms (e.g., being overly concerned with which leg to lead with when starting to walk), thereby introducing more obstacles into his everyday life. His condition has worsened rapidly in the last few years. These problems had caused his mother much suffering when she took him on outings. He received assistance from his mother in all domains of functioning in his daily life. He was very anxious, and dependent on his mother, which made it difficult for him to be separated from her. He visited a clinic regularly, but had no medical illnesses that caused psychological effects. He was enrolled in the treatment program due to his mother’s wish to improve this situation.

His Vineland-II evaluation showed low level of adaptation and presence of marked maladaptive behavior (**Tables 1, 2**).

**Table 2:** Indices of pre- and post-treatment maladaptive behaviors and internalizing problems.

Participant	Index	Pre-treatment		Post-treatment	
		score		score	
Ken	Maladaptive Behavior Index	19	Elevated	17	Normal
	Internalizing	21	Clinically significant	17	Normal
Atsushi	Maladaptive Behavior Index	23	Clinically significant	22	Clinically significant
	Internalizing	23	Clinically significant	22	Clinically significant

### Overview of Interventions

We conducted two overnight treatment programs, with the first one consisting of 15 sessions over 6 days and 5 nights and the second one consisting of 10 sessions over 4 days and 3 nights. Both programs were certified by the Japanese Association of Rehabilitation Psychology. The therapist was a male certified clinical psychologist and was certified as a therapist by the Japanese Association of Rehabilitation Psychology.

During the Dohsa-hou sessions, relaxation was used to ease the physical and psychological tension of participants, thereby reducing anxiety that might hinder action. While these activities reduce physical tension, altering participants’ psychological experiences is also important, i.e., feeling changes in the level of tension in their own bodies and in their movements (Fujino, 2013). Dohsa-hou tasks include walking and balancing in the standing position, actions that participants find difficult to initiate and control, due to excess tension. These tasks lead to a feeling of greater control over one’s own movements and a heightened sense of self-efficacy. Among the experiences that clients encounter are situations in which they are supported and their non-verbal intentions are understood (Naruse, 1995). Intention is an essential component in Dohsa-hou to develop agency (Naruse, 2000). This element of the program leads to the psychological changes that define the aim of the intervention (Naruse, 1997). In addition to participating in three daily 50-min sessions (except first and last days), clients also participate in daily group therapy (a group of five or six participants), which includes interventions that incorporate play and self-expression activities. Some of these activities are designed to help participants learn to make decisions by themselves and to express their opinions. Further details of the program have been previously published (Naruse, 1995; Imura and Chervenkova, 2016).

### Treatment Course

The goal of physical relaxation was to reduce the tension in the client’s lower back, hip joint, upper back, and shoulders. To achieve this goal, the client worked interactively with the therapist on tasks to ease tension, gain body control and to accumulate experiences through such interactions to be able to facilitate future involvement with others. During these interventions, the therapist supported the client’s efforts, aware of his role in providing the client with a sense of accomplishment at being able to achieve goals on his own and thereby improving his self-efficacy.

#### Case 1 (Ken)

Ken had a high level of tension in his lower back and could not control his back or hip joints very well when balancing between the left and right sides of his body; therefore, it was difficult for him to maintain his balance when standing on one foot. There was also some tension and stiffness in his upper back and shoulders. He responded to tasks presented by the therapist, but rarely initiated them himself.

We conducted a total of 15 sessions. At first, Ken passively followed the therapist’s lead, and it was difficult for him to actively participate in the tasks. However, the tension in his upper and lower back gradually eased, and as the therapist established rapport while ensuring that his anxiety around the therapist had abated, the therapist saw him gradually try to perform the tasks actively, begin to smile and show more emotion in his facial expressions, and with this attitude change, even enjoy performing the tasks. Initially, he was reluctant to participate in group therapy, but gradually started to actively participate on his own and enjoyed interaction with other participants.

By the end of the program, he was more active when performing tasks, and in his everyday life, he was no longer relying on his mother to make decisions and choices for him. His anxiety decreased and we saw changes in his relationships with others as well; for example, he was engaging with others on his own more often. One month after completing the program, his score on the Maladaptive Behavior Index of the Vineland-II have significantly improved (**Table 2**). His clinically significant internalizing problems and elevated maladaptive behavior had lessened to normal levels. His mother also reported that his maladaptive behaviors decreased in comparison to the period prior to participation in the program.

#### Case 2 (Atsushi)

Due to very high tension and no flexibility in the lower back, Atsushi was not able to control his upper body very well when in a sitting or standing position. His shoulders and upper back were also very tense, and he could often be seen sitting or standing very still, with his shoulders slouched and in a tense posture. When the therapist asked him to perform the tasks, he was able to do them once, but then he gave up performing them immediately after completion.

We conducted a total of 10 sessions. At first, he was so anxious that he often cried out because of his concerns about the other participants, and he often became distracted. However, as he participated in additional sessions, he gradually learned how to relax his shoulders and lower back through his own movements. His upper back was still very tense and could not be relaxed unless the therapist actively assisted with its movement. As his tension began to ease, he began to participate actively in the outings and group activities that were part of the program. The compulsive behaviors exhibited previously during movement also decreased gradually and he was able to move smoothly from one location to another.

By the end of the program, the initial lack of flexibility caused by high tension had significantly decreased. Remarkably, he responded more often to engagement by those around him and learned to interact with others without hating it.

One month after completing the program, slight changes were observed in his maladaptive behaviors and internalizing problems (**Table 2**). Although his level of maladaptive behaviors remained at a clinically significant level, there were reductions in the severity of problematic behaviors, such as in his dependence on his mother, feelings of anxiety, sleep disturbances, and compulsive-like behavior. His mother reported that his social interactions had changed considerably (the compulsive behaviors that persisted were not as severe), and that supporting him had become easier.

## Discussion

This short-term intervention program improved emotional problems in two adults with Down syndrome. Maladaptive behavior generally reduces as they age; however, internalizing problems (e.g., depression and anxiety) are maintained, or slightly increased, in young adults (Dykens et al., 2002; Foley et al., 2015). The reason why these types of problems are prominent in Down syndrome is not well clarified, but several factors have been noted to enhance risk for such issues (Prasher, 2002; Capone et al., 2006; Akahoshi et al., 2012; Dekker et al., 2015). Psychological and emotional responses are one of the core components of the problems. Therefore, interventions targeting these issues are needed to reduce symptoms and enhance well-being in adults with Down syndrome and their family (Dykens, 2007).

The current intervention focused on reduction of high levels of tension, reduction of anxiety in social and interpersonal situations, and improving a sense of self-efficacy in the clients. High levels of physical tension were loosened in the Dohsa-hou sessions, accompanied by a reduction of anxiety. Psychological changes accompanied with the relaxation experience could be a core component of therapeutic improvement observed as a result of Dohsa-hou treatment, although physical relaxation alone has an important role in improving anxiety (Fujino, 2016). Also, a recent study showed that relaxation intervention improved anxiety and self-esteem in adults with intellectual disabilities (Bouvet and Coulet, 2016). In addition to the Dohsa-hou sessions, group therapy provided opportunities to express feelings and to practice self-directing behavior in interpersonal situations. The results suggest that these experiences contribute to reduce anxiety in social situations, which leads to decreased compulsive (routinized) behavior. Several studies suggest that routinized behaviors play a functional role in anxiety reduction (Glenn and Cunningham, 2007; Glenn and Nananidou, 2016), thus, reduction of anxiety is a contributing factor for reduced routinized behavior. In Dohsa-hou sessions and group therapies, participants achieve relaxation, standing, and communication tasks with therapists, which may influence improvement in their self-efficacy. Increasing a sense of self-efficacy is a protective factor for depressive disorders (Alesi et al., 2015). Improved postural stability by relaxation and standing tasks may also be associated with the noted improvement in adaptation and psychological distress, as suggested in previous studies (Adachi, 2015; Fujino and Imura, 2015). As several experimental studies suggested associations between postural stability and psychological state (Stambolieva and Angov, 2010; Coelho and Balaban, 2015), improved postural stability by relaxation and standing tasks may also be associated with the noted improvement in adaptation and psychological distress (Adachi, 2015; Fujino and Imura, 2015). Even postural stability is considered as an essential factor affecting psychological state in Dohsa-hou (Naruse, 1973, 1995), how postural stability affect psychological state is not well investigated. Further research is needed to clarify the therapeutic mechanisms of those interventions.

In addition to those internal factors, functional impairment in older people with Down syndrome could be affected by limited social services and attitudes of family members as suggested in previous studies (Bertoli et al., 2011; Covelli et al., 2015). Support from family members helps their daily living and participation in activities; however, attitudes of family members could interfere their autonomy in a certain situation (Covelli et al., 2015). Assessing those factors could be helpful in understanding individual problems when we evaluate their clinical conditions.

### Complicating Factors

Several factors may affect improvement of psychological problems in a Dohsa-hou program. The two participants differed in degree of improvement after completion of the program. While Ken improved significantly and maladaptive symptoms decreased to normal levels, Atsushi showed some improvement, but substantial symptoms remained stable. The reason for such a disparity could be due to individual differences in level of adaptation, as well as severity of psychological problems prior to the intervention. Additionally, prior research has shown that autistic traits in Down syndrome may affect severity of compulsive repetitive behaviors and adaptive functioning (Carter et al., 2007; Powell et al., 2017), which may adversely influence efficacy of the treatment.

## Concluding Remarks

This is one of few psychosocial approaches that has been proposed to improve psychiatric and internalizing problems in young adults with Down syndrome. This case report suggests that Dohsa-hou could be an effective therapeutic approach for internalizing problems in adults with Down syndrome. Improvement in internalizing symptoms may be affected by the strengthening of self-efficacy and improvement in communication with the therapist. Dohsa-hou has beneficial effects toward emotional and psychological distress in adults with Down syndrome, although improvement may be affected by the baseline severity of the emotional and psychological problems prior to treatment.

## Author Contributions

The HF confirms being the sole contributor of this work and approved it for publication.

## Conflict of Interest Statement

The author declares that the research was conducted in the absence of any commercial or financial relationships that could be construed as a potential conflict of interest. The reviewer VC and handling Editor declared their shared affiliation, and the handling Editor states that the process nevertheless met the standards of a fair and objective review.

## Abbreviations

Vineland-II: Vineland Adaptive Behavior Scales-II
